# Flow resistance characteristics of the stem and root from conifer (*Sabina chinensis*) xylem tracheid

**DOI:** 10.1371/journal.pone.0259117

**Published:** 2021-10-28

**Authors:** Tianyu Xu, Shuteng Zhi, Ennan Zheng, Chengcheng Yan

**Affiliations:** 1 School of Hydraulic and Electric Power, Heilongjiang University, Harbin, China; 2 School of Traditional Chinese Medicine, Zhejiang Pharmaceutical College, Ningbo, China; University of Alberta, CANADA

## Abstract

Xylem tracheids are the channels for water transport in conifer. Tracheid flow resistance is composed of tracheid lumen resistance and pit resistance. The single tracheid structure parameters in the stem and root of *Sabina chinensis* were obtained by dissociation and slicing, combined with numerical simulation to analyze the tracheid flow resistance characteristics. The results showed that the tracheid lumen resistance was determined by the tracheid width and tracheid length. The pit resistance was determined by the number of pits and single pit resistance. The single pit resistance was composed of four elements: the secondary cell wall, the border, the margo and the torus. The margo contributed a relatively large fraction of flow resistance, while the torus, the border and the secondary cell wall formed a small fraction. The size and position of the pores in the margo had a significant effect on the fluid velocity. The number of pits were proportional to tracheid length. The power curve, S-curve and inverse curve were fitted the scatter plot of total pit resistance, total resistance, total resistivity, which was found that there were the negative correlation between them. The three scatter plot values were larger in the stem than in the root, indicating that the tracheid structure in the root was more conducive to water transport than the stem. The ratio of tracheid lumen resistance to pit resistance mainly was less than 0.6 in the stem and less than 1 in the root, indicating that the pit resistance was dominant in the total resistance of the stem and root.

## Introduction

Conifers are one of the largest biological communities in the world, and their flow of xylem depends on the adjacent tracheids [1, 2]. The restriction imposed by the hydraulic path is considered to be the major constraint on the maximum heights attainable by conifers [3], Tracheids are short conduits relative to vessels, and each single tracheid has tens to hundreds pit structures [4, 5], which connect the tracheids to form the xylem water transport channels. The flow inside the tracheids mainly depends on the tracheid lumen and pit structure [6, 7]. The pit structure in many conifers is of great significance because the pit membrane prevents the spread of embolism [8, 9]. Indeed, the contribution of tracheid lumen to flow resistance has been largely ignored in describing the water movement of plants’ tracheids [10, 11].

The pit resistance is more difficult to measure or model because the pit magnitude is between micrometers and millimeters and the structure is complex [12, 13]. The computational fluid dynamics (CFD) method is used to analyze the flow mechanism inside the pit structure. Valli et al. [14] used the Lattice-Boltzmann method to simulate the flow resistance of the pits, and the margo was regarded as a group of randomly oriented fibers, thereby obtaining the relationship between the pressure drop and porosity. Chen Q et al. [15] used a low Reynolds number k-ε model and a porous medium model to numerically simulate the change in the pit structure, and found that the flow resistance coefficient of the pits was inversely proportional to the pit diameter, pit aperture diameter and porosity.

A number of theoretical and empirical data showed that the tracheid lumen resistance should play a prominent role in determining the efficiency of water supply to the xylem. Tracheids have wider lumen to reduce flow resistance. The tracheid lumen resistance appears to constitute less 50% of the total flow resistance for the xylem [8]. However, Lancashire et al. [16] showed a “typical” tracheid and calculated that the pit resistance accounted for 29% of the total resistance.

In order to further study the relationship between the tracheid lumen resistance and pit resistance, Sabina chinensis was taken as the research sample, and anatomical experiments were arranged to obtain tracheid lumen structures and pit structure parameters, combined with numerical simulations to establish a mathematical model for calculating the single-tracheid resistance. It can: (1) obtain the relationship among the tracheid lumen parameters (tracheid length, tracheid width and number of pits) in the stem and root; (2) evaluate the resistance components and internal flow characteristics of pit model; (3) analyze the total resistance, total pit resistance and total resistivity of single tracheid in the stem and root. The results will provide a reference for the flow characteristics of different single-tracheid structures and promote a deeper understanding of water transport in the plant xylem.

## Materials and methods

### Experimental materials

**Fig 1:**
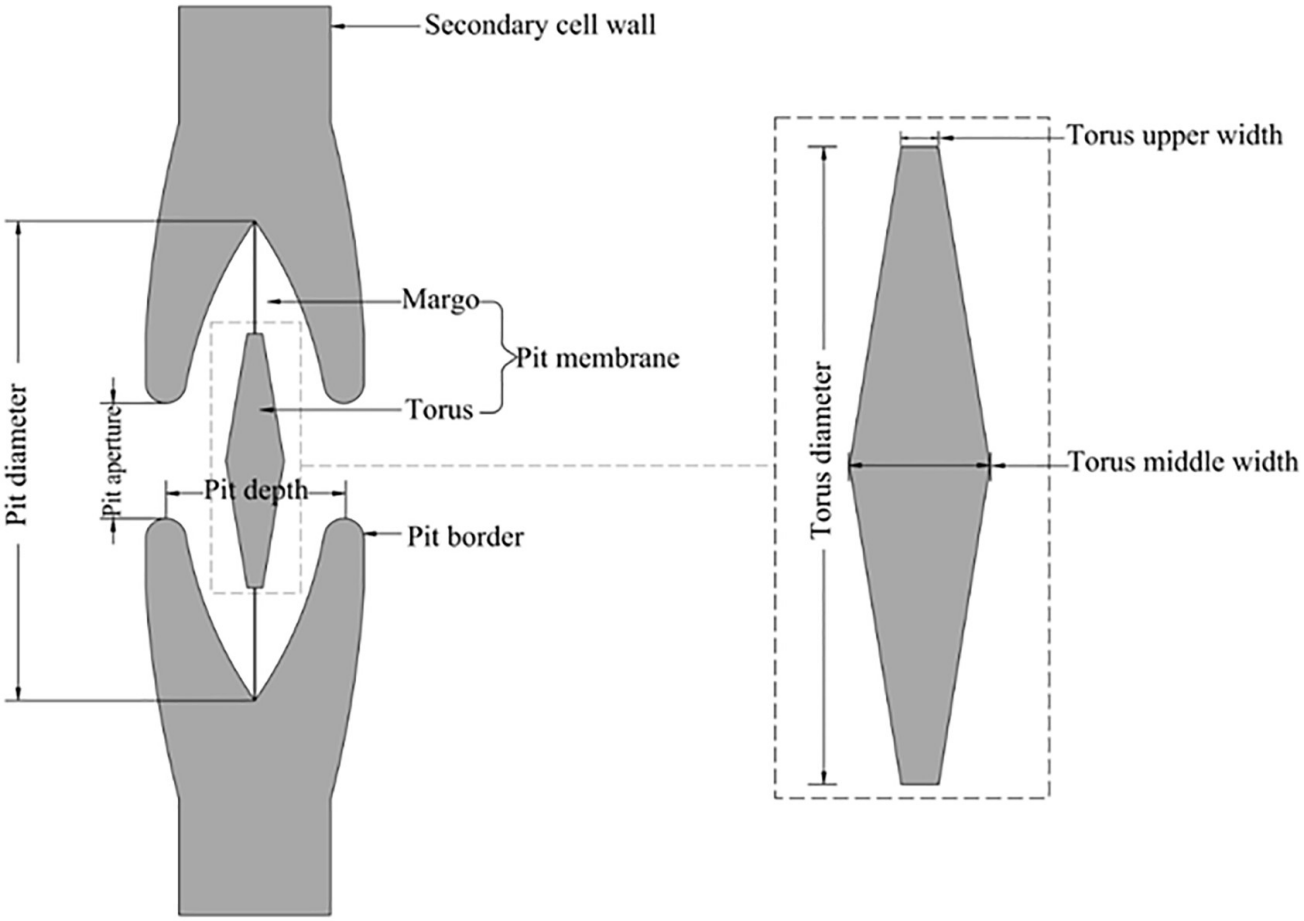
Schematic diagram of torus-margo bordered pit structure.

In our study, the *Sabina chinensis* was sampled at the Kunming University of Science and Technology in Yunnan Province, the southwest of China, which was located 24°84′50″N and 102°86′49″E at an elevation of 1860 m above sea level. The growth area had a subtropical-plateau mountain monsoon climate, and the annual temperature difference varies little. With sufficient sunshine, the annual average temperature was 15°C, the annual average rainfall was approximately 732 mm, and the frost-free period was more than 240 d. The stem samples were collected from the middle part of the stem. The root samples were excavated from the base of the trees and sampled downward along the outward roots until reaching the target diameter (0.8–1.0 cm). The 4–6 samples of roots and stems were collected, sliced within 3–4 days and analyzed by scanning electron microscopy and digital microscope. Fig 1 showed the terms of the torus-margo bordered pit structure (TPS).

### Slice preparation

The slice processing in this experiment adopted the method of Xu [17] experiment. Sawing the rhizome blocks: taking the root and stem blocks of the *Sabina chinensis* (0.7 cm×0.7 cm×0.7 cm), the cross section, tangential section and radial section were repaired under the dissecting microscope.

#### Wood softening

The water-boiling method was used to soften the rhizome blocks of the *Sabina chinensis*. The repaired blocks were put into a beaker filled with distilled water, and the beaker was vacuumed in a vacuum pump until the blocks sank to the bottom. The blocks were taken out and put into a pressure cooker for approximately 3–5 h until they were fully softened.

#### Slicing

The slicer was mounted on the microtome, tightened and slightly tilted. The degree of inclination depended on the hardness of the wood. The angle between the side of the experimental slicer and the surface of the wooden block was approximately 10°. The thickness of the slice was generally 5 to 15 μm. The cross sections were perpendicular to the longitudinal axis, the tangential section)s were parallel to the wood ray, and theradial section)s were perpendicular to the wood ray. After the sectioning was completed, the sections were removed from the slicer with a brush and transferred to a petri dish of water. Three pieces of each surface were cut for spares.

#### Sealing

The cover glass and glass slide were washed with alcohol before sealing, the slices were placed on the glass slide, and a layer of transparent agent was evenly applied on the slices (alcohol-glycerol 1:1). The cover glass was gently pressed onto the glass slide, and the bubbles were slowly pushed out.

#### Labeling

Label the upper left side of the slides, then put them into the slice box for storage.

### Tracheid dissociation

Prepare thin root and stem strips of the *Sabina chinensis*, 1.5 cm long and match- shaped, by a cutter. Make a mixture by the ratio of 1:1 with 30% hydrogen peroxide and glacial acetic acid on the experimental operation platform in the fume hood.

The strips and the mixture were put into a test tube. The upper end of the test tube was fixed on the iron stand while the bottom end was placed in a water bath to sufficient dissociation, processed 2–3 h at 95°C, till the strips turned white. After the test tube was cooled, the treatment solution should be gently washed with distilled water, and repeated several times. The test tube with the appropriate amount of distilled water was shaken to fully separate the structure of the strips, and the glycerol-alcohol (1: 1) transparent liquid was used to make the isolated temporary pieces. The dissected tracheids were observed by digital microscope. 50 groups of tracheids were randomly selected, and their tracheid length, tracheid width, and number of pits were measured respectively.

### Scanning electron microscope observation

**Fig 2:**
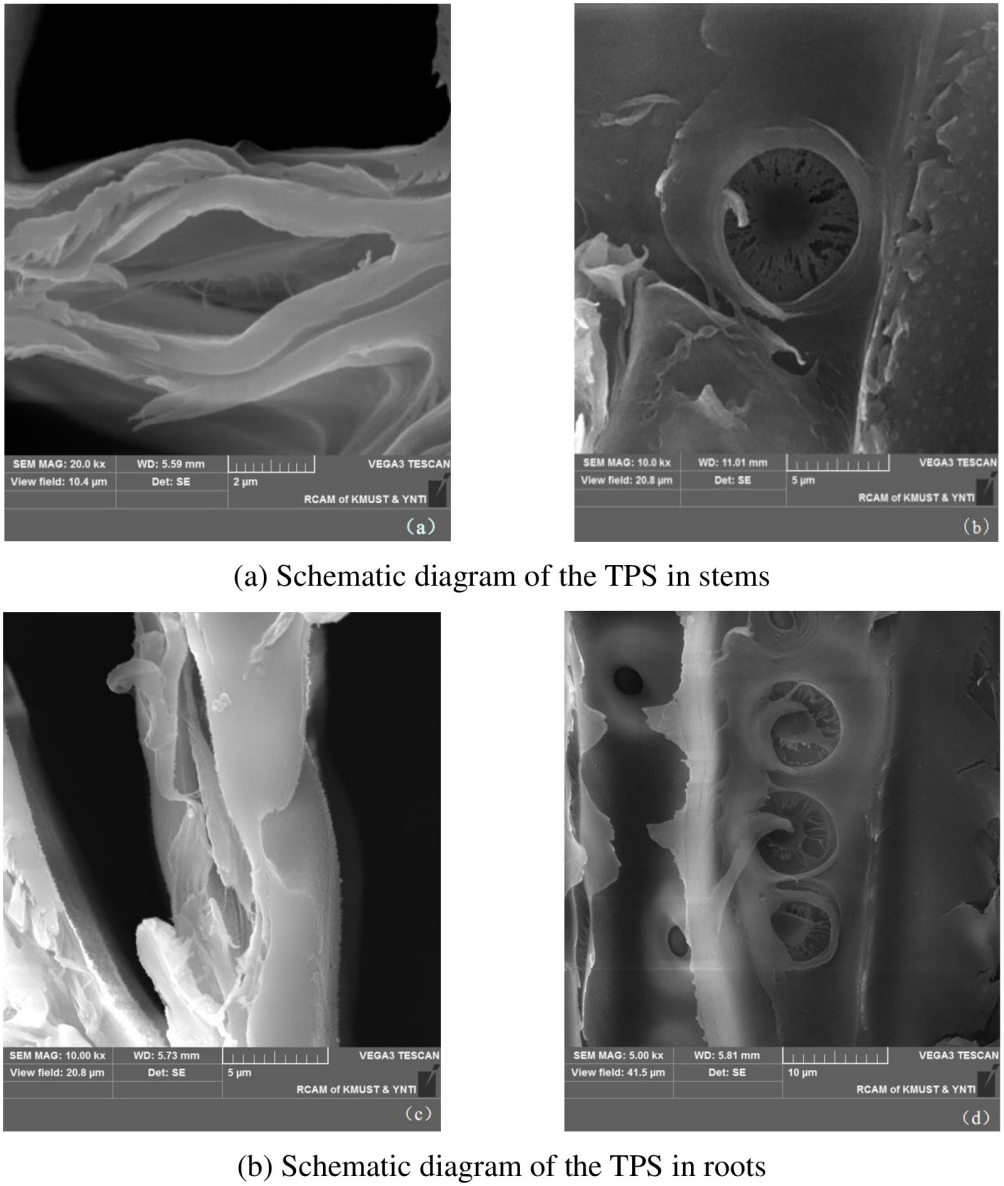
Schematic diagram of the TPS structure of the *Sabina chinensis*. (a) Schematic diagram of the TPS in stems. (b) Schematic diagram of the TPS in roots.

The sealed samples were soaked in distilled water and the glycerin was dislodged by multiple cleanings, then the samples were put into 30%, 50%, 70% and 90% ethanol solutions (30 minutes) to dislodge the moisture. Finally, the samples were put into a 100% ethanol solution for an hour, with gold spraying of the samples after air drying for at least 12 hours. The samples were observed by a scanning electron microscope (SEM) at the Analytic and Testing Research Center of Yunnan. The cross sections of the samples were used to observe the torus thickness and pit width (Fig 2a and 2c). The tangential section)s and radial sections of the samples were used to observe the pit diameter and pit aperture size (Fig 2b and 2d).

### Model construction of TPS

**Fig 3:**
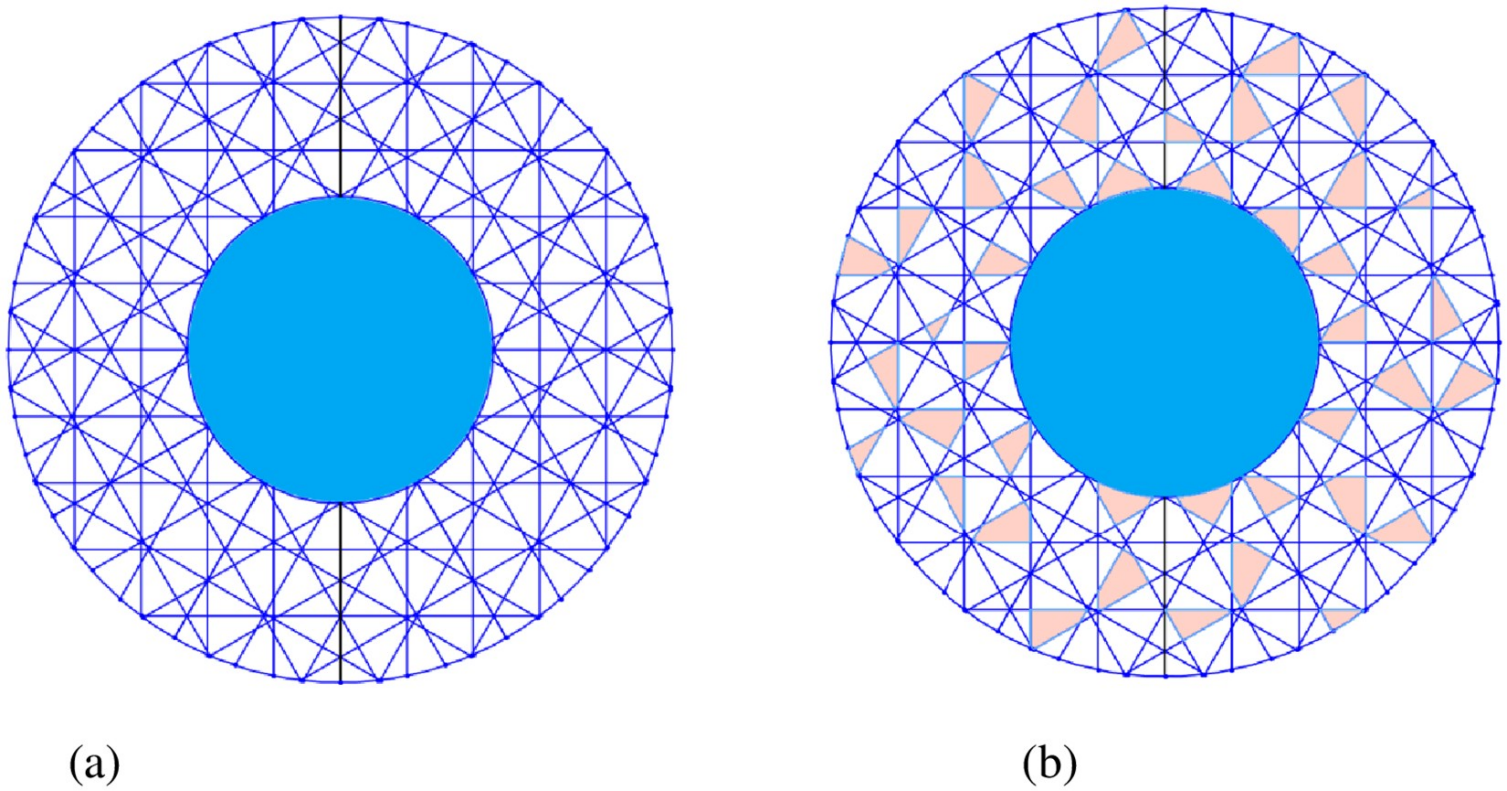
Schematic diagram of pit membrane of the TPS. (a) Pit membrane structure. (b) porosity distribution.

The detailed structure parameters of the TPS were shown by the scanning electron microscopy images. The water transport characteristics were analyzed by the computational fluid dynamics method. Combined with the anatomical observation and model construction, and considered the permeability of the pit membrane, the expression of the pit membrane referred to in the literature [7] and the structure was shown in Fig 3a. In our study, the pit membrane permeability was 40% by anatomical observation (Fig 3b).

**Fig 4:**
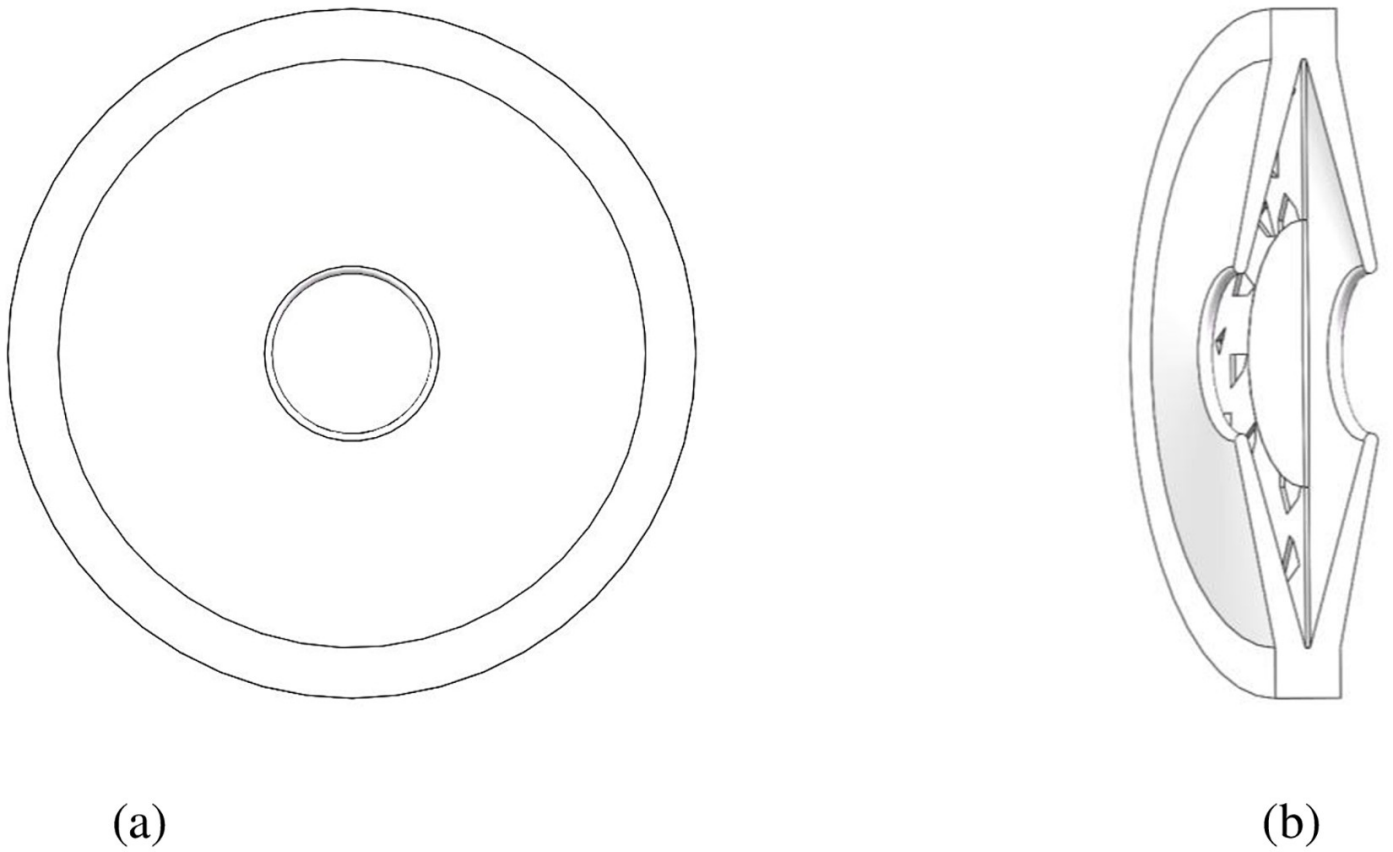
Schematic diagram of the TPS model. (a) Front view of pit structure. (b) Sectional view of pit structure.

Using Solid-Works, a complete three-dimensional model of the TPS was established (Fig 4).

**Fig 5:**
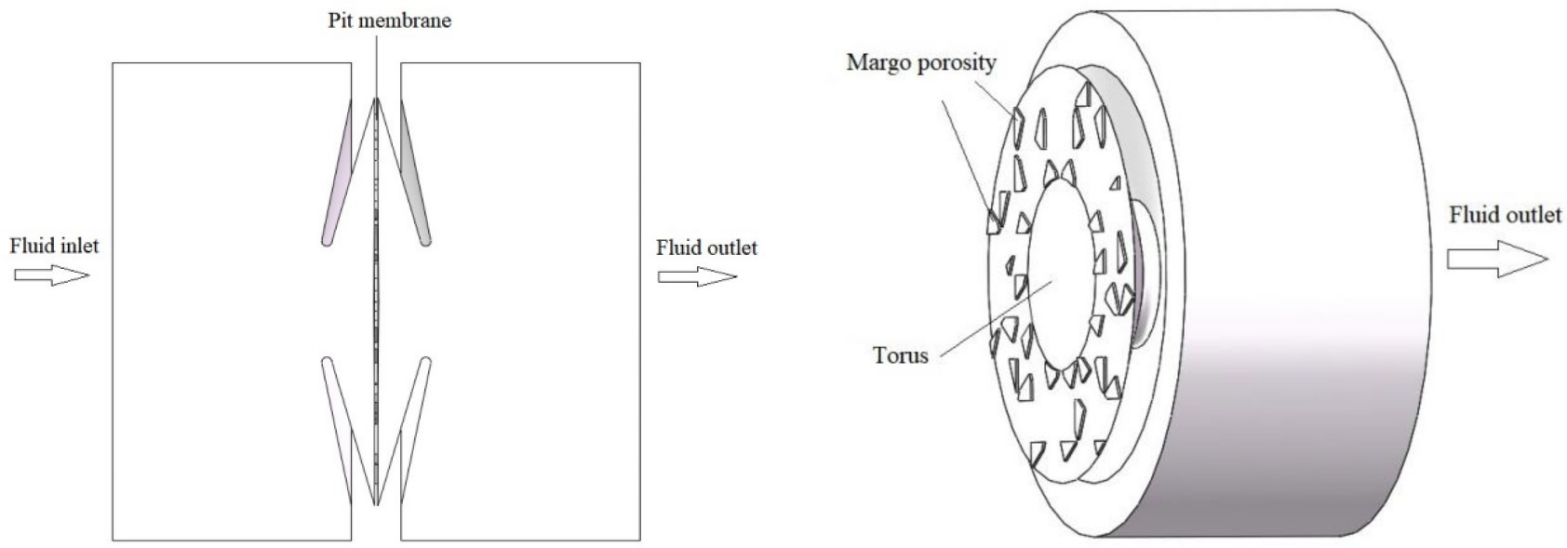
Fluid domain calculation model of TPS.

**Fig 6:**
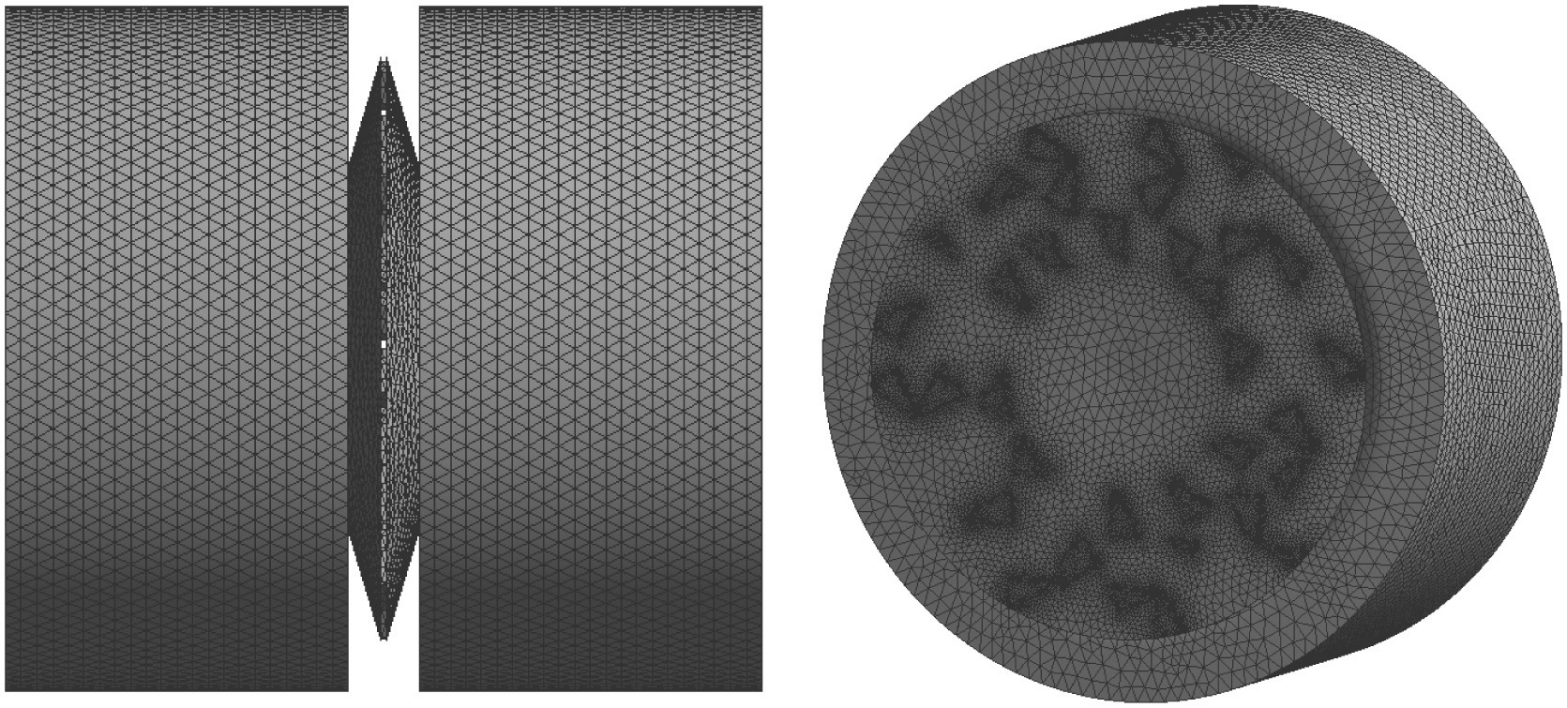
The mesh of the TPS.

The simulation of the model was a fluid domain, and the fluid was water. For the boundary conditions, the pressure was zero at the model outlet, and the flow velocity was 0.1 mm/s at the model inlet (Fig 5). Considered the irregularity of the TPS, the grid generation used the non-grid structure of the tetrahedron and hexahedral. The maximum and minimum of the unit size were 4.8×10^-7^m and 4.8×10^-9^m, respectively. In this part, the scale of the mesh is based on the prediction accuracy of the inlet/outlet pressure drop, and the mesh size independence test is performed (Table 1). The pressure drop difference between the standard mesh and the fine mesh is 0.28%. The mesh number had no effect on the calculation results, so the standard mesh number was used, and the total number of mesh in the model was approximately 401,345 (Fig 6). The PowerCube-S01 with a high-performance computing system was used for the simulation.

**Table 1:** Mesh size independence test.

	Mesh number	Pressure drop difference
Coarser	114521	--
Coarse	193542	1.25%
Standard	401345	0.46%
Fine	1025632	0.28%

### Mathematical model of xylem single tracheid

**Fig 7:**
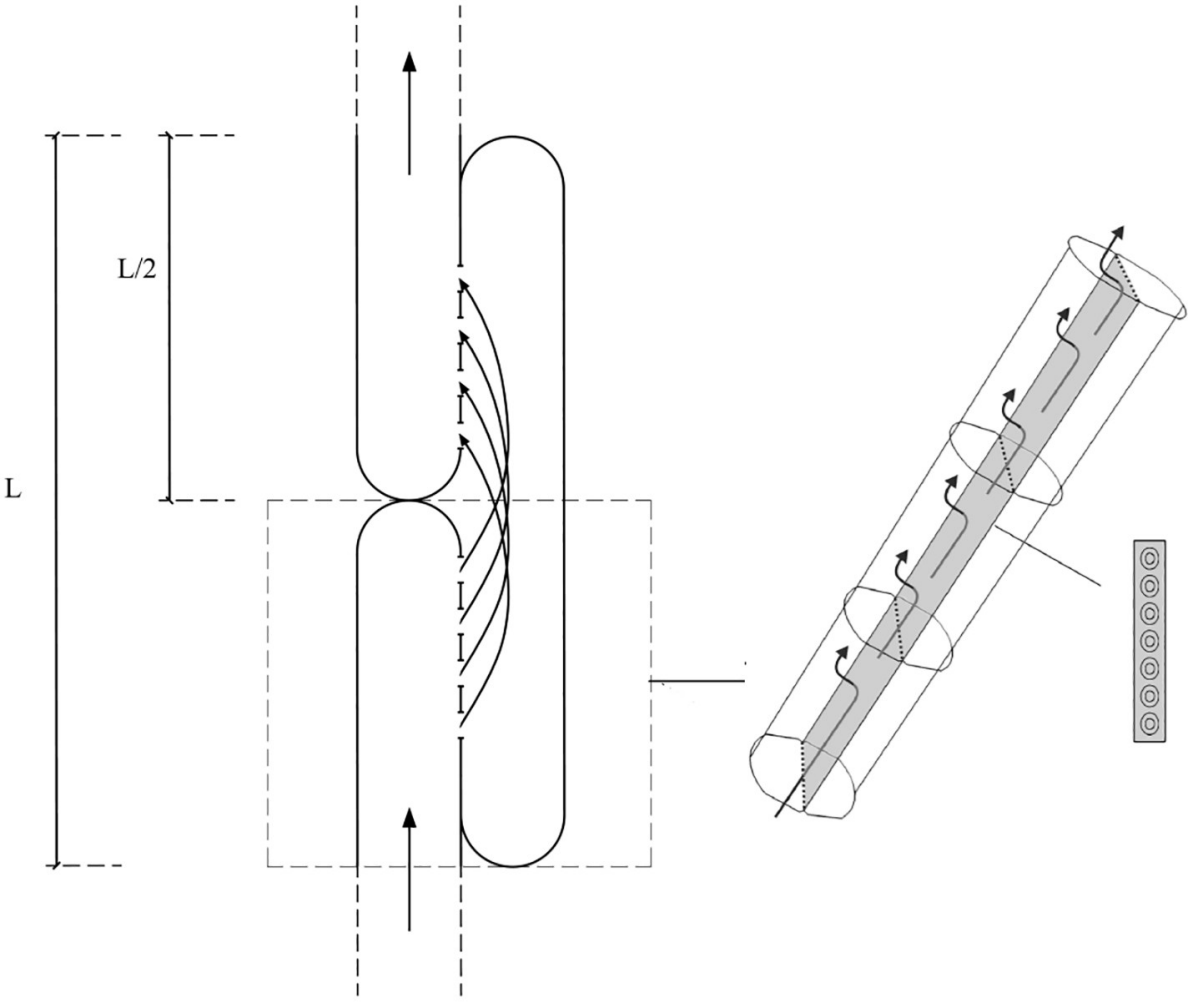
Model of tracheid water flow.

According to experimental anatomy, the torus-margo bordered pits in the tracheid junction were distributed in a straight line along the tracheid axis, and the tracheid connection mode was shown in Fig 7.

When water moves through the xylem tracheid, the water enters the bottom half of the single tracheid through the pit structure, flow upward through the tracheid, and finally leaves through the pit structure in the upper half of the single tracheid. Because the pits are evenly distributed on the tracheid wall, the distance that water flow in the tracheid is the half of the tracheid length. Thus, water flowing through the single tracheid encounters two part resistances, which is tracheid lumen resistance and pit resistance. The tracheid lumen resistance (*R*_*l*_) can be described by Hagen Poiseuille equation and was given by the expression

$$R_{l}=\frac{128\mu }{\pi D^{4}}\times \frac{L}{2}$$ (1)

where *L* is the tracheid length, *D* is tracheid diameter and *μ* is the water dynamic viscosity.

The single pit resistance was *R*_*ind*_, and the total pit resistance of the single tracheid was given by the expression

$$R_{p}=2R_{ind}/M$$ (2)

where *M* is the number of pits in the single tracheid. The total resistance (*R*_*tot*_) is given by the expression

$$R_{tot}=R_{l}+R_{p}=\frac{64\mu L}{\pi D^{4}}+\frac{2R_{ind}}{M}$$ (3)

So the total resistivity (resistance per unit length) was given by the expression

$$R_{res}=\frac{R_{tot}}{L}=\frac{64\mu }{\pi D^{4}}+\frac{2R_{ind}}{ML}$$ (4)

## Results

### Anatomical differences between stems and roots

Based on the microscopic images of the different sample sections, the structural parameters of the TPS in the roots and stems were measured. The measurements were taken for each character listed in Table 2.

**Table 2:** Structural parameters of the TPS in roots and stems.

Structural parameters	Stem	Structural parameters	Root
Pit diameter	8.96μm	Pit diameter	15.71μm
Torus diameter	4.14μm	Torus diameter	7.14μm
Torus upper width	0.11μm	Torus upper width	0.13μm
Torus middle width	0.26μm	Torus middle width	0.29μm
Pit aperture diameter	2.79μm	Pit aperture diameter	4.28μm
Pit depth	3.04μm	Pit depth	3.81μm
Secondary cell wall height	1.15μm	Secondary cell wall height	1.34μm
Secondary cell wall width	1.54μm	Secondary cell wall width	1.92μm

The pit diameter of the roots was 1.75 times than that of the stems, the pit aperture diameter of the roots was 1.53 times than that of the stems, the pit depth of the roots was 1.25 times than that of the stems, the torus diameter of the roots was 1.72 times than that of the stems, the torus upper width of the roots was 1.18 times than that of the stems, the torus middle width of the roots was 1.12 times than that of the stems, the height of the secondary cell wall of the roots was 1.17 times than that of the stems, and the width of the secondary cell wall of the roots was 1.25 times than that of the stems.

### Velocity distribution of fluid in the TPS

**Fig 8:**
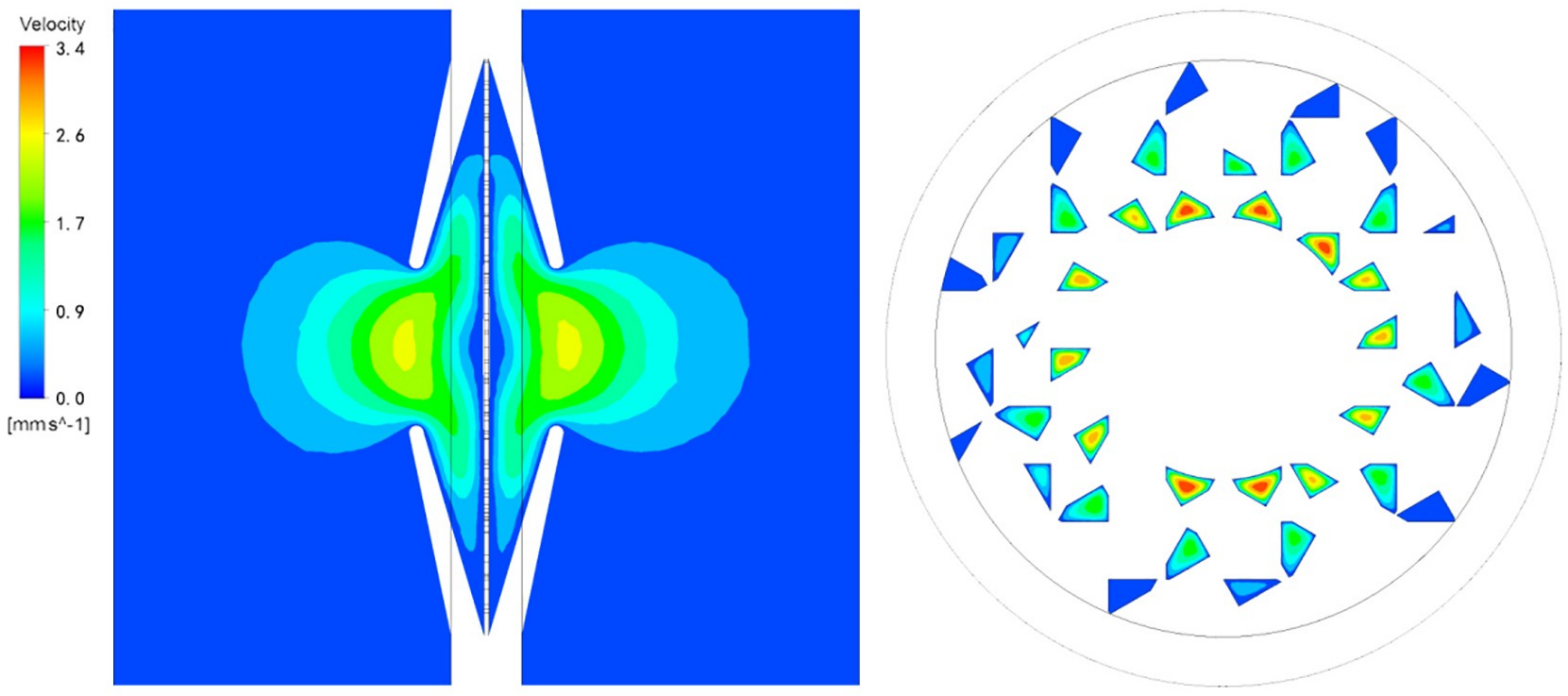
Velocity distribution of fluid in the torus-margo bordered pit.

Fig 8 showed the flow characteristics of root models, and the stems models reflected similar flow sections within the pit structure. The high flow velocity region was occurred in the narrow pit aperture. The fluid could not pass the middle of the pit cavity and was forced to move to both ends of the torus. the inner and outer of the torus were connected to the margo, so the fluid could just flow the pores of the margo. Within an individual pit, flow velocity was strongly affected on the margo pores. The higher flow velocity region was obtained at the larger pores in the margo, and the flow velocity was zero at the pores where the torus was further away. At the same distance from the torus, the large pores in the margo had higher velocity. The fluid flowing through the pit was blocked by the front border and torus, which caused the fluid to suddenly contract and shunt. The shunt fluid will turn sharply through the margo and the back border, and finally converge at the back pit aperture. As a result, the fluid velocity changed significantly, leading to the local energy loss.

### Resistance components of the TPS

Via the numerical simulation, the inlet and outlet pressure and the average flow rate (*q*) were obtained. The total pressure drop (*Δp*) is the pressure difference between the outlet and inlet of the TPS model. The flow resistance (*R*_*ind*_) of the TPS was also calculated according to the total pressure drop (*Δp*) divided by the average flow rate (*q*).

For the purpose of estimating the roles of various TPS components, such as the secondary cell wall, pit border, the torus and the margo, in the initial calculation, models were constructed with the secondary cell wall, and then with the pit border, the torus, and finally the margo added to the model, which was called model 1, model 2, model 3 and model 4 respectively. *Δp* and the *R*_*ind*_ are shown in Table 3. Then, according to Table 4, the resistance ratio of each component in the TPS was obtained.

**Table 3:** Flow resistance of TPS in the stems and roots.

models	stem			root		
	Δp/Pa	q/(m^3^·s^–1^)	R_ind_/(Pa·s·m^-3^)	Δp/Pa	q/(m^3^·s^–1^)	R_ind_/(Pa·s·m^-3^)
model 1	0.68	9.94×10^−15^	0.68×10^14^	0.36	2.65×10^−14^	1.36×10^13^
model 2	22.66	9.94×10^−15^	2.28×10^15^	15.90	2.65×10^−14^	5.89×10^14^
model 3	52.31	9.94×10^−15^	5.26×10^15^	53.61	2.65×10^−14^	2.02×10^14^
model 4	134.17	9.94×10^−15^	1.35×10^16^	100.34	2.65×10^−14^	3.79×10^15^

**Table 4:** Resistance calculation method.

Calculation method	Formula	Calculation method	Formula
Secondary cell wall flow resistance	R_s_ = R_1_	Fraction due to secondary cell wall	F_1_ = R_1_/R_4_
Border flow resistance	R_b_ = R_2_-R_1_	Fraction due to pit border	F_2_ = R_2_-R_1_/R_4_
Torus flow resistance	R_t_ = R_3_-R_2_	Fraction due to Torus	F_3_ = R_3_-R_2_/R_4_
Margo flow resistance	R_m_ = R_4_-R_3_	Fraction due to Margo	F_4_ = R_4_-R_3_/R_4_
Total flow resistance	R_tot_ = R_4_		

Note: R_1_ is the flow resistance of the root and stem of the model 1; R_2_ is the flow resistance of the root and stem of the model 2; R_3_ is the flow resistance of the root and stem of the model 3; R_4_ is the flow resistance of the root and stem of the model 4.

It can be seen from complete pit structure (model 4) that the difference of *Δp*, *q* and *R*_*ind*_ resulted from the difference of pit size between the roots and stems. *Δp* of the complete pit structure in roots was reduced by 25.21%, *q* was increased by 166.60%, and *R*_*ind*_ was decreased by 71.93% compared to the stems. *R*_*ind*_ of roots was lower than that in the stems.

The model 1 in Table 3 shows very low pressure drop (*Δp*) and flow resistance (*R*_*ind*_) because only the secondary wall structure was added. With pit border, the torus and the margo components were added to the model, the pressure drop (*Δp*) and flow resistance (*R*_*ind*_) increased significantly. The sum of *R*_*ind*_ (the secondary cell wall and the pit border) was a relatively small component in the roots and stems of the *Sabina chinensis*, accounting for 16.89% and 15.54% of the total flow resistance, respectively. However, the torus and the margo were dominant in the total flow resistance. *R*_*ind*_ of the torus in the roots and stems accounted for 22.07% and 37.76% of the total flow resistance, respectively, and *R*_*ind*_ of the margo in the roots and stems accounted for 61.04% and 46.70% of the total flow resistance, respectively (Table 5).

**Table 5:** Fraction of resistance to different components of TPS in the stems and roots.

Pit component	Fraction due to stem	Fraction due to root
Secondary cell wall	0.50%	0.36%
Border	16.39%	15.18%
Torus	22.07%	37.76%
Margo	61.04%	46.70%

Although the difference of pit size between the roots and stems was large, the proportion of the components was similar, in which the resistance ratio of the margo was the largest, followed by the torus and the pit border, and the secondary cell wall was the smallest.

### Correlation resistance analysis of single tracheid structure

#### The relationship among the tracheid structural parameters

**Fig 9:**
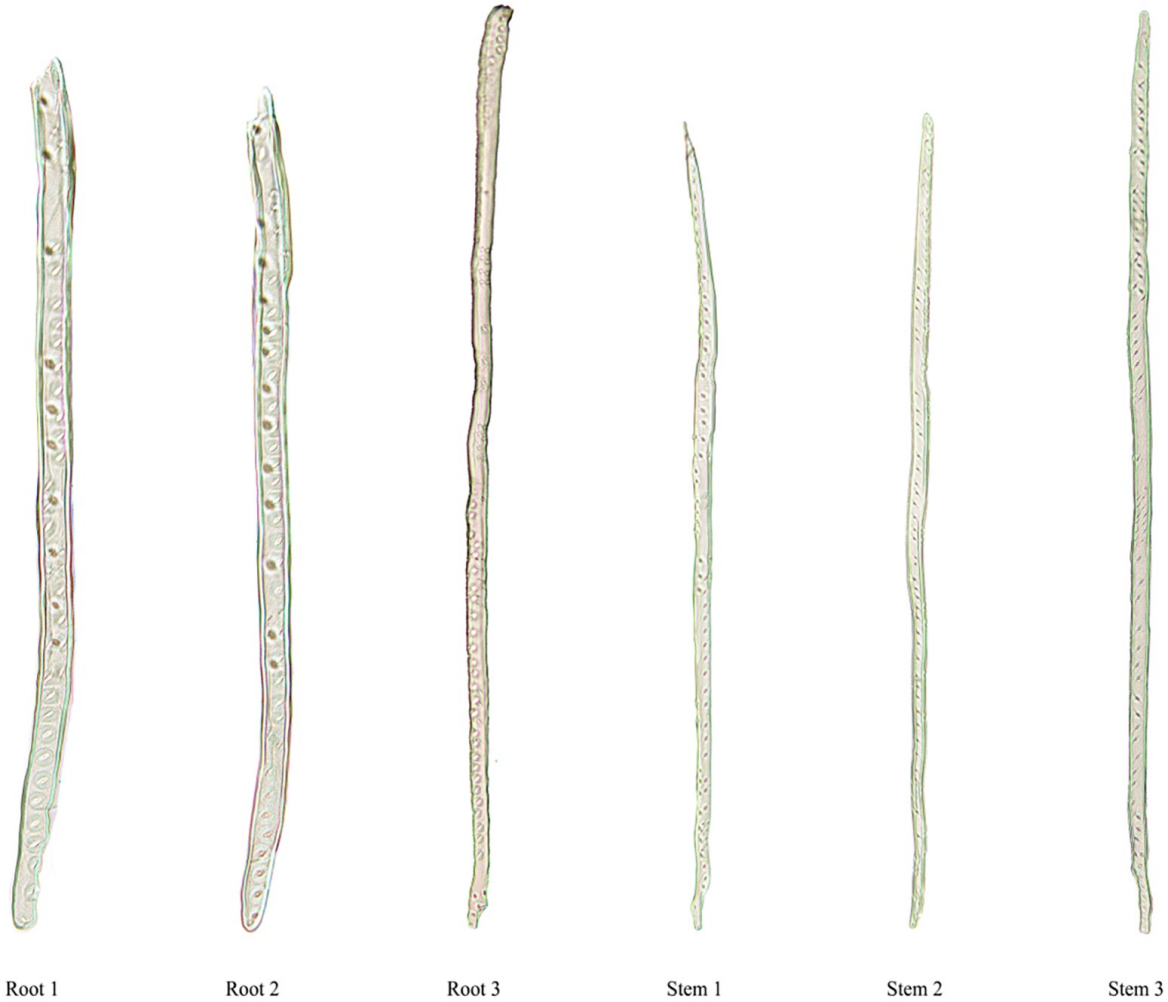
Schematic diagram of the single tracheid structure.

Tracheid structure parameters were obtained by dissociating root and stem of the *Sabina chinensis* (50 groups per part), including tracheid length, tracheid width, and number of pits. The tracheid structure of the stems and roots of the *Sabina chinensis* was shown in Fig 9.

**Fig 10:**
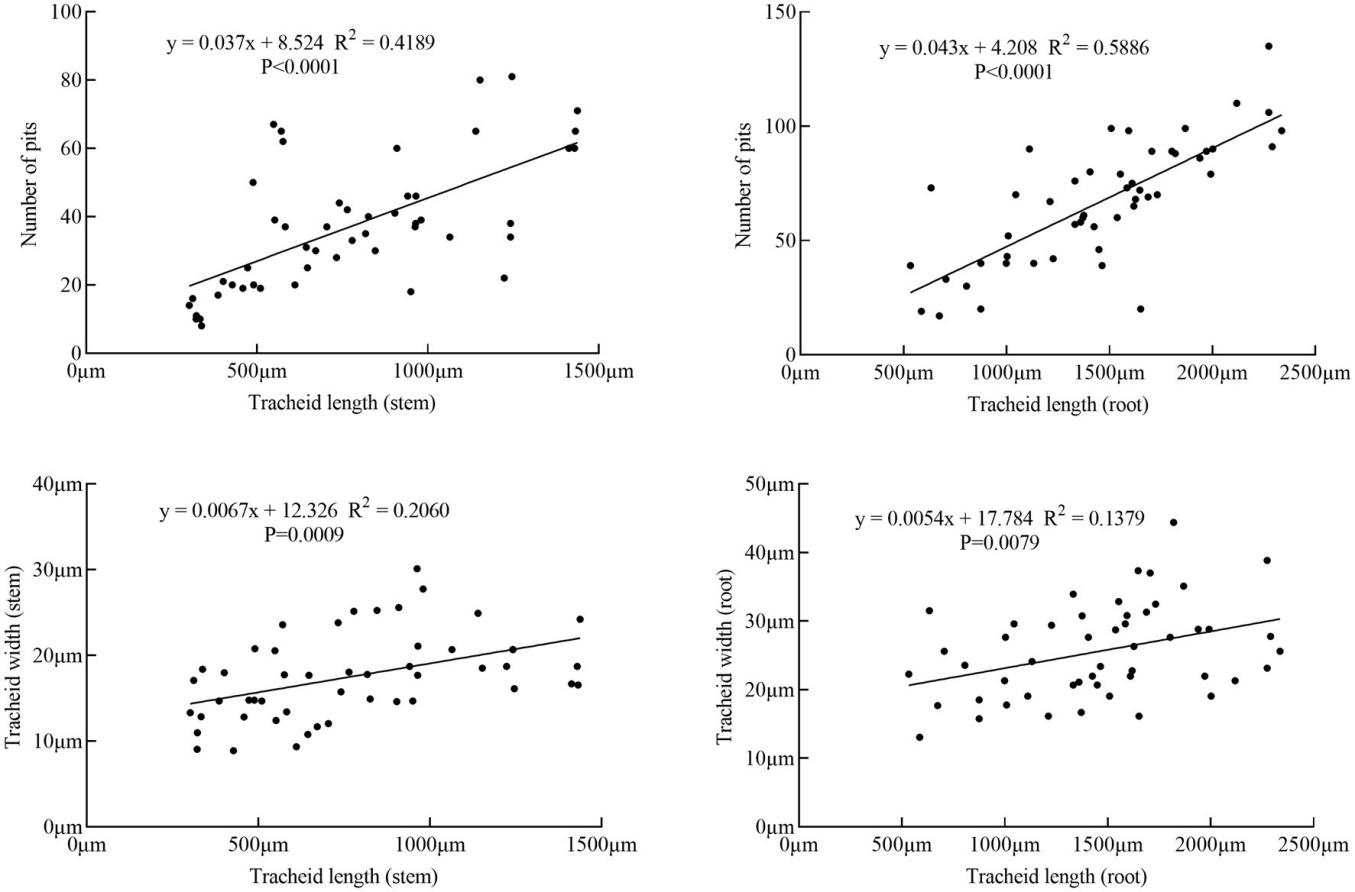
Scatter plot of tracheid length, tracheid width and number of pits.

The tracheid length in root was between 500μm and 2500μm, the tracheid length in stem was between 300μm and 1500μm; The tracheid width in root was mainly between 18μm and 34μm, the tracheid width in stem was mainly between 10μm and 25μm; The number of pits in root was mainly between 25 and 110, the number of pits in stem was mainly between 10 and 70. It can be seen from the Fig 10 that the tracheid width and the number of pits of the single tracheid in the root and stem increased with an increase in the tracheid length. Pearson correlation analysis was performed on the structural parameters of single tracheids. The results showed that there was a significant correlation between tracheid length and number of pits in the root and stem of the Sabina chinensis. There were significant differences in tracheid length and number of pits, tracheid length and tracheid width (P<0.01).

#### The relationship between resistance parameters and tracheids length

**Fig 11:**
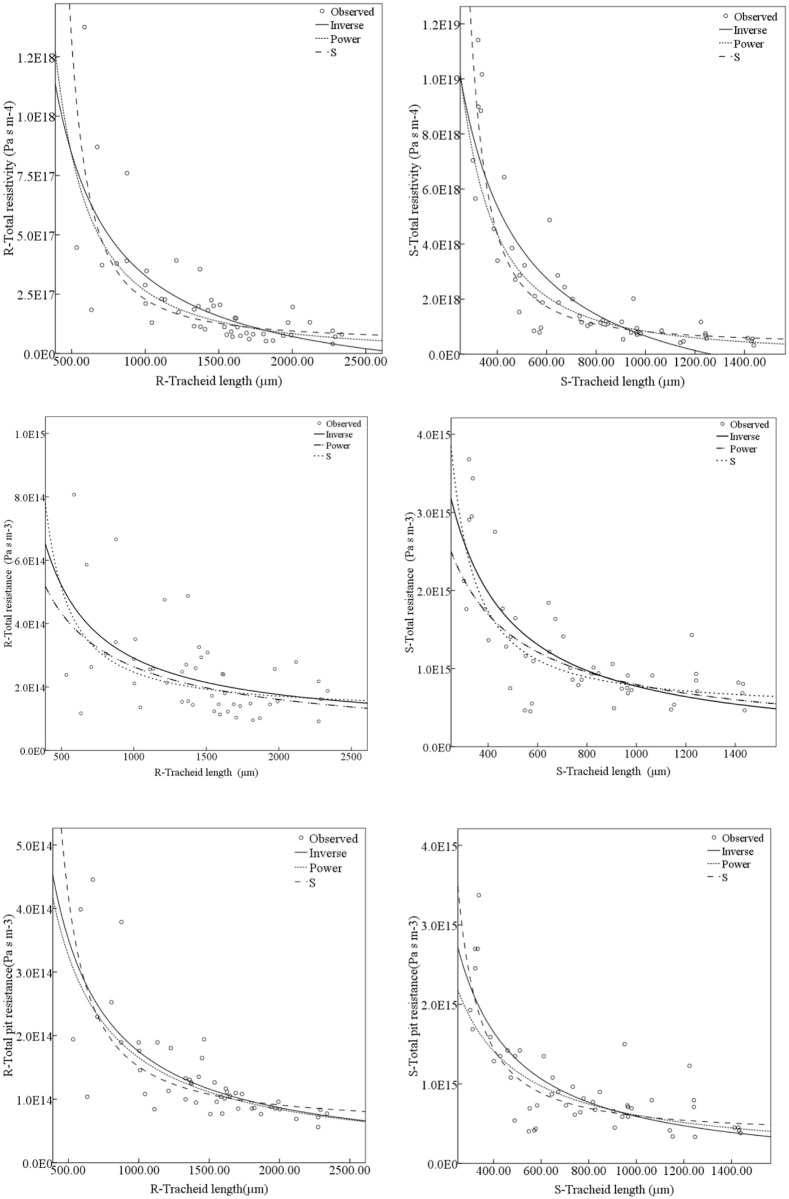
The relationship between total pit resistance, total resistivity and tracheids length. (a) Scatter plot of tracheid length and total pit resistance. (b) Scatter plot of tracheid length and total resistance. (c) Scatter plot of tracheid length and total resistivity.

The total pit resistance, total resistance and the total resistivity of the single tracheid were calculated according to the formula (2), formula (3) and formula (4). The relationship between total pit resistance, total resistance, total resistivity and tracheids length was shown in the Fig 11. It can be seen from the scatter plot that the total pit resistance values, total resistance values and total resistance values of the single tracheid in the stem were higher than that of the root. The total pit resistance, total resistance and total resistivity in the root and stem decreased with an increase in the tracheid length.

The Power curve, S curve and Inverse curve were fitted to the scatter plot (Fig 11a–11c), and the optimal fitting equation was determined by determination coefficient (R^2^) and significance (sig.). The results were shown in Table 6. In the stem and root, the significance of the three fitting curves was less than 0.000, indicating that the tracheid length had a significant correlation among the total pit resistance, the total resistance and the total resistivity. The Inverse-curve had the highest determination coefficient in the total pit resistance scatter plot of the stem, and the Power-curve had the highest determination coefficient in the total pit resistance scatter plot of the root, both indicating that the fitting degree was the best. The Inverse-curve had the highest determination coefficient in the total resistance scatter plot of the stem and root, indicating that the fitting degree was the best. The S-curve had the highest determination coefficient in the total resistivity scatter plot of the stem, and the power-curve had the highest determination coefficient in the total resistivity scatter plot of the root, indicating that the fitting degree was the best.

**Table 6:** Correlation coefficient of total pit resistance, total resistance and total resistivity.

Scatter plot	Curve fitting	Fitting equation(stem)	R^2^	Sig.	Fitting equation(root)	R^2^	Sig.
Tracheid length and total pit resistance	S-Curve:	$\text{ln}y=33.438+\frac{587.349}{x}$	0.588	0.000	$\text{ln}y=31.630+\frac{1018.598}{x}$	0.572	0.000
	Power-Curve:	*y* = −0.921ln *x* + 3.530 × 10^17^	0.538	0.000	*y* = −0.979ln *x* + 1.433 × 10^17^	0.628	0.000
	Inverse-Curve:	$y=\frac{7.078\times 10^{17}}{x}\text{-}1.\text{164}\times 10^{14}$	0.632	0.000	$y=\frac{1.767\times 10^{17}}{x}\text{-}1.856\times 10^{12}$	0.534	0.000
Tracheid length and total resistance	S-Curve:	$\ln y=33.758+\frac{532.023}{x}$	0.550	0.000	$\ln y=32.408+\frac{731.916}{x}$	0.226	0.000
	Power-Curve:	*y* = −0.826ln *x* + 2.387 × 10^17^	0.490	0.000	*y* = −0.714ln *x* + 3.671 × 10^16^	0.256	0.000
	Inverse-Curve:	$y=\frac{8.0\times 10^{17}}{x}\text{-}2.55\times 10^{13}$	0.608	0.000	$y=\frac{2.296\times 10^{17}}{x}+6.142\times 10^{13}$	0.268	0.000
Tracheid length and total resistivity	S-Curve:	$\text{ln}y=40.138+\frac{1112.755}{x}$	0.812	0.000	$\text{ln}y=38.214+\frac{1755.126}{x}$	0.615	0.000
	Power-Curve:	*y* = −1.814ln *x* + 2.284 × 10^23^	0.802	0.000	*y* = −1.663ln *x* + 2.581 × 10^22^	0.656	0.000
	Inverse-Curve:	$y=\frac{3.145\times 10^{21}}{x}\text{-}2.488\times 10^{18}$	0.760	0.000	$y=\frac{5.136\times 10^{20}}{x}\text{-}1.853\times 10^{17}$	0.552	0.000

**Fig 12:**
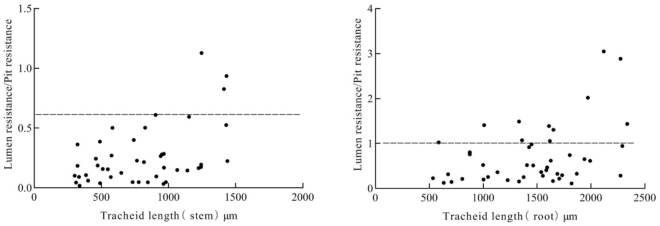
Scatter plot of the ratio of the tracheid lumen resistance to pit resistance.

Compared with the total resistance (Fig 11b) and the total pit resistance (Fig 11a) scatter plot, the resistance power magnitude of the ordinate had not changed. The ratio of lumen resistance to pit resistance was shown in Fig 12, which was mainly less than 0.6 in the stem and was less than 1 in the root.

## Discussion

The results showed that the flow resistance of the TPS in the roots was significantly lower than that in the stems. The reason was that the roots had a wider and longer tracheid from the larger TPS size and margo area [18]. Though anatomical observations, the pores on the pit membrane were distributed randomly, thus the pit membrane structure was complicated [4]. Lancashire^16^ and Chen Qi et al. [15] used the uniform mesh or homogeneous medium as the model boundary to construct the pit membrane. In our research, with the method of Hacke et al. [7] to construct the pit membrane, it was found that the most obvious area of flow velocity change was the pores in the margo, indicating that the pores in the margo had a great influence on the total flow resistance. The pores in the margo were far from the torus, and the axial distance between the border and the margo was shorter, resulting in a high shear force in the fluid, causing the fluid flow velocity to decrease. Therefore, the area of the pores in the margo, which was away from the torus, was not conducive to water transport [7, 13].

The two models (stem and root) showed many similarities in terms of the flow resistance inside the TPS. The numerical simulation was used to obtain the flow resistance of each component in the pit structure, where the secondary cell wall and the pit border accounted for 15.54%-16.89% of the flow resistance in the roots and stems, and similar trends were recorded by the Choat et al. [10]. The flow resistance of the pit membrane accounted for 83.11%-84.46% of the total resistance. The reason was that the fluid moved toward both ends of the torus due to the torus hinder and flowed through the pores in the margo [13, 18], and the flow velocity was higher in the pores of the margo and near torus area, producing more local energy loss. Certainly, another important function of the pit membrane was that when the tracheid was filled with air, the torus moved to one side to fit the pit aperture and sealed the TPS to prevent the spread of an embolism [19].

In this paper, a single tracheid calculation model was established, and 50 groups of single tracheids were analyzed by combining the lumen resistances and pit resistances. The scatter plot of tracheid length and total resistance, total pit resistance and total resistivity were fitted by three methods (power curve, S curve and inverse curve), and was found in a certain tracheid length, the total resistance, total pit resistance and total resistivity asymptote tend to be parallel, and the further reduction of the three parameters was the smallest [9, 20], which demonstrated that the lumen resistance and pit resistance restrict the tracheid length, tracheid width, and number of pits, because the tracheid structure should maintain the balance between flow resistance and embolism vulnerability [19]. Larger pit structures are beneficial to reducing the flow resistance of tracheids inside the xylem, which will also increase the possibility of embolism occurring between tracheids [21, 22]. The greater the proportion of transport capacity will be lost if the vessel becomes embolized in the longer and wider single tracheid. Hacke et al. [23] and Lancashire [16] reported that pit resistance and lumen resistance were co-limiting the tree xylem water transport capacity, and the total resistance was the smallest when the ratio of the two resistances was 1: 1. In this paper, through the analysis of the ratio of lumen resistance to pit resistance, it was found that the ratio mainly was less than 0.6 in the stem, indicating that the pit resistance was dominant in the stem [24]. In the root, the ratio of the two parameters had increased, but the ratio was mostly less than 1. The reason was that the number of pits on a single tracheid in the root and stem of the *Sabina chinensis* was arranged in a straight line, resulting in a small number of pits, leading to large pit resistance ratio large.

## Conclusions

The torus-margo bordered pit structure of root and stem was similar in the *Sabina chinensis*, while the size was different. The flow resistance of the margo was the largest, then the torus, and the lowest was the border. The highest flow velocity region in the TPS was obtained at the maximum pore of the margo near the torus. The number of pits were proportional to tracheid length. The total resistance, total pit resistance and total resistivity were inversely proportional to tracheid length, and the three parameters were larger in the stem than that of the root. The pit resistance was dominant in the total resistance of the stem and root. In the scatter plot of tracheid length and total pit resistance, the inverse curve fitted best in the stem and the power curve fitted best in the root. In the scatter plot of tracheid length and total resistance, the inverse curve fitted best in the stem and root. In the scatter plot of tracheid length and total resistivity, the S curve fitted best in the stem and the power curve fitted best in the root.

## Supporting information

S1 Table 50 groups of tracheids structural parameters. (PDF)
