# Supplementary material for: Flow resistance characteristics of the stem and root from conifer (Sabina chinensis) xylem tracheid
Source: PLoS One. 2021 Oct 28;16(10):e0259117. doi: 10.1371/journal.pone.0259117 (PMC8553130; doi:10.1371/journal.pone.0259117)
Supplement: S1 Table — (PDF) [file pone.0259117.s001.pdf]

Table 1. 50 groups of tracheids structural parameters

Root	length/μm	width/μm	number	Stem	length/μm	width/μm	number
1	634.27	31.51	73	1	401.36	17.98	21
2	1538.53	28.74	60	2	333.42	12.85	10
3	875.14	18.50	40	3	472.69	14.77	25
4	2290.56	27.75	91	4	459.14	12.81	19
5	1332.21	33.93	57	5	322.38	9.05	10
6	1360.81	21.09	58	6	323.14	10.99	11
7	1449.69	20.68	46	7	337.92	18.38	8
8	1651.67	16.15	20	8	301.88	13.31	14
9	705.92	25.59	33	9	311.72	17.08	16
10	1733.16	32.47	70	10	764.71	18.05	42
11	1375.25	30.75	61	11	950.72	14.67	18
12	1211.88	16.16	67	12	611.34	9.35	20
13	534.07	22.28	39	13	386.12	14.67	17
14	1009.20	17.79	52	14	489.03	14.78	50
15	2274.28	38.86	106	15	551.73	12.43	39
16	2336.77	25.59	98	16	576.65	17.76	62
17	876.36	15.79	20	17	548.34	20.56	67
18	673.72	17.67	17	18	583.12	13.42	37
19	587.00	13.08	19	19	644.37	10.78	31
20	1044.54	29.62	70	20	427.86	8.88	20
21	1464.53	23.40	39	21	510.13	14.67	19
22	1938.99	28.81	86	22	704.19	12.04	37
23	1372.21	16.68	60	23	672.23	11.68	30
24	805.71	23.58	30	24	570.95	23.58	65
25	1226.96	29.40	42	25	1246.56	16.12	81
26	1991.96	28.81	79	26	1438.08	24.21	71
27	1688.62	31.30	69	27	1140.77	24.91	65
28	1647.68	37.37	72	28	647.92	17.67	25
29	1618.18	22.75	65	29	941.17	18.73	46
30	1593.06	30.80	98	30	962.86	30.12	37
31	1406.43	27.64	80	31	846.65	25.25	30

32	2119.27	21.30	110	32	741.51	15.75	44
33	1584.75	29.62	73	33	1431.78	16.55	65
34	1868.71	35.10	99	34	1429.28	18.73	60
35	1820.77	44.38	88	35	1242.29	20.68	38
36	2002.59	19.07	90	36	1242.04	20.68	34
37	1610.25	21.99	75	37	826.62	14.92	40
38	999.49	21.30	40	38	817.73	17.79	35
39	1627.00	26.33	68	39	1224.00	18.73	22
40	1971.40	21.99	89	40	909.27	25.59	60
41	1332.89	20.68	76	41	903.62	14.63	41
42	1426.24	21.99	56	42	1152.82	18.50	80
43	1133.22	24.12	40	43	980.29	27.75	39
44	2274.28	23.13	135	44	1412.60	16.68	60
45	1508.79	19.07	99	45	490.45	20.79	20
46	1804.03	27.64	89	46	1064.95	20.68	34
47	1003.32	27.64	43	47	965.58	21.09	46
48	1553.72	32.87	79	48	964.84	17.67	38
49	1706.40	37.02	89	49	733.04	23.83	28
50	1111.53	19.07	90	50	778.47	25.16	33
